# En bloc pelvic resection for advanced ovarian cancer preceded by central ligation of vessels supplying the tumor bed: a description of surgical technique and a feasibility study

**DOI:** 10.1186/s12957-016-0894-5

**Published:** 2016-04-29

**Authors:** Jacek Jan Sznurkowski

**Affiliations:** Department of Surgical Oncology, The Medical University of Gdańsk, ul. Smoluchowskiego 17, 80-214 Gdańsk, Poland

**Keywords:** Ovarian cancer, En bloc pelvic resection, Complete pelvic peritonectomy, No-touch isolation, Surgery, Cytoreduction

## Abstract

**Background:**

The resection of all visible malignancies increases the likelihood for long-term survival in epithelial ovarian cancer. The complete extinguishment of pelvic disease is possible using en bloc pelvic resection. The no-touch isolation technique aims to reduce cancer cells flowing from the primary tumor site to the liver and other organs by ligating blood and lymphatic vessels first. objectives are to present the operative details and to establish the feasibility of the modified technique of en bloc pelvic resection, which begins with the central ligation of vessels supplying the tumor bed.

**Methods:**

Twenty patients with pelvic tumor extensively infiltrating into adjacent pelvic organs were uniformly operated on. The surgical plan commenced with incisions along the lateral peritoneal reflections immediately medial to the white line of Toldt followed by a retroperitoneal central ligation of ovarian and mesenteric vessels and the ovarian lymphovascular flow. Then, the routine steps of en bloc pelvic resection were performed. Data on treatment were assessed.

**Results:**

In all cases, no gross residual disease was achieved. The median durations of the surgical procedure and the hospital stay were 320 min (range: 205–430 min) and 12 days (range: 7–44 days), respectively. The complications were as follows: wound infection (*n* = 1), anastomosis dehiscence (*n* = 1), total parenteral nutrition (*n* = 4), and death (*n* = 1, PE). The median follow-up time period was 19 months (range: 8–31 months). No patient experienced a recurrence of pelvic disease.

**Conclusions:**

Performing a central ligation of vessels supplying the tumor bed prior to an en bloc pelvic resection is feasible with acceptable morbidity and mortality rates.

## Background

Epithelial ovarian carcinoma (EOC) is the leading cause of death due to gynecologic malignancy, and more than 60 % of women with newly diagnosed EOC present with advanced stage disease [1]. Current standard treatment for advanced ovarian cancer consists of a primary debulking surgery (PDS) followed by platinum-based chemotherapy [2, 3]. Several studies support a greater survival rate in patients who were optimally cytoreduced compared with patients who were suboptimally cytoreduced [4–7].

The possibility of excising all visible lesions (*R* = 0, microscopic) is crucial for deciding whether to perform an extensive cytoreductive surgery.

En bloc pelvic resection is effective for achieving maximal cytoreduction while eliminating the pelvic disease in advanced primary EOC patients with extensive pelvic organ involvement including viscera [8].

Lymphovascular ligation before tumor manipulation during cancer resection is termed the “no-touch isolation” technique [9]. This technique aims to reduce the intraoperative dissemination of cancer cells and was proposed by Barnes [10] to decrease the incidence of liver metastases by diminishing the intraoperative dissemination of colorectal cancer cells.

Animal studies have confirmed this reduction [5], and recent data in humans suggest a trend towards reduced tumor cell dissemination with the no-touch isolation method [11, 12]. The benefit of this method in terms of improved patient survival, however, remains unproven [9].

To achieve cytoreduction to microscopic residual disease in disseminated EOC cases with peritoneal and visceral metastases consistent with the requirements of cancer no-touch isolation, a modification of the en bloc pelvic resection procedure was introduced.

This modification aims initially to reach the retroperitoneal space where the lymphatic and vascular systems supplying the cancer tissue are located. Next, a central vascular ligation is performed. Finally, the intraperitoneal cancer compartment (including the infiltrated portion of bowel) is surrounded from the outside (healthy tissues) by separating the involved peritoneum from the bladder and the pelvic wall and then resected in one block. A colorectal anastomosis is created.

This paper aims to present a new surgical approach for advanced EOC and to share the initial experience of applying this approach.

## Methods

### Population

From May 2013 to October 2014, a modified en bloc pelvic resection was performed on 20 patients with advanced EOC at the Department of Gynecologic Oncology, Oncology Center, in Red Cross Hospital in Gdynia, Poland. The study was approved by the local Ethics Committee (NKBBN/263/2012).

Inclusion criteria are as follows: age younger than 80 years old, ASA performance status 0 to 2 [13], resectable disease evaluated by computed tomography (CT) scan and/or positron emission tomography (PET), no significant co-morbidities, and provided informed written consent. Exclusion criteria are as follows: other malignant pathologies, an extra-abdominal metastasis, a complete intestinal obstruction, or active infections.

The treatment plan consisted of performing a central vascular ligation prior to an en block pelvic resection. This treatment was a component of primary surgery aiming to remove all visible disease using different types of peritonectomy with related resections defined by Sugarbaker (Table 1) [14]. Each patient was operated on by JJS, who designed the surgical plan. The observation period ended 1st October 2015.

**Table 1:** The visceral resections and parietal peritonectomy procedures

Type of peritonectomy	Provided resections
Pelvic peritonectomy	Uterus, ovaries, and rectosigmoid colon
Left upper quadrant peritonectomy	Greater omentectomy and spleen
Right upper quadrant peritonectomy	Tumor on Glisson’s capsule of the liver
Anterior parietal peritonectomy	Old abdominal incisions, umbilicus, and epigastric fat pad
Omental bursectomy	Gallbladder and lesser omentum

### Surgical plan

1.  Mobilization of the left and right colons
  The peritoneum is incised along the lateral peritoneal reflections immediately medial to the white line of Toldt from the left and right paracolic sulci cephalad to the splenic and hepatic flexures of the colon. The mesentery of the left and right colons is divided from its retroperitoneal attachments (Fig. 1a).

**Fig. 1:**
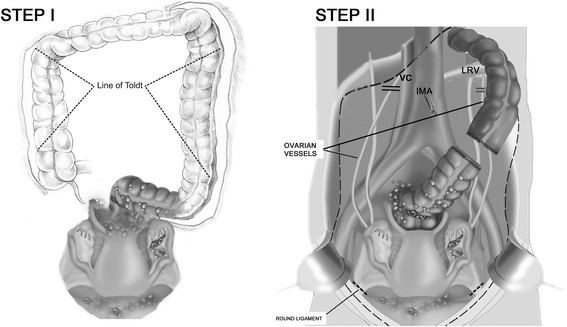
The crucial steps of the proposed modification. **a** Entry into the extraperitoneal space and mobilization of the left and right colons. **b** Central vascular ligation of the tumor-bearing segment
  Both ureters and the plane over Gerota’s fascia are identified to avoid compromising the kidneys.
2.  Dissection and central ligation of ovarian vessels
  The left and right ovarian vessels are dissected and ligated at the level of the left renal vein and the vena cava, respectively (Fig. 1b).
3.  Central lymphovascular ligation with retroperitoneal selective lymph node dissection (if necessary)
  The lymphovascular flow is ligated by several clips. Enlarged, suspicious lymph nodes are excised at the level of the left renal vein (LRV) down towards the pelvis in one block. Where possible, a nerve-sparing technique is used.
4.  Central vascular ligation of the tumor-bearing segment of the colon
  The inferior mesenteric artery (IMA) is ligated on the aorta. The artery’s individual branches are then resected as they arise from this vascular trunk (Fig. 1b). The Y-configuration of the left colic and sigmoidal vessels is converted to a V-configuration to preserve the intermediate arcade. The inferior mesenteric vein (IMV) is divided as it courses around the duodenum.
5.  Division of the colon*
  The colon is dissected 5 cm beyond the boundary of the cancer. A previous central vascular ligation and the mobilization of the left colon allowed for packing all the viscera, including the proximal colon, into the upper abdomen.
6.  Dissection of the mesorectum 2–3 cm below the retroperitoneal flexure
7.  Stripping of the vesical peritoneum with the underlying fatty tissues from the surface of the bladder
  The point of tissue transection is precisely located between the bladder musculature and its adherent fatty tissue with peritoneum. The inferior limit of the dissection is the anterior wall of the vagina.
8.  Stripping of the parietal peritoneum
  The peritoneal incision around the pelvis is connected to the peritoneal incisions in the right and left paracolic sulci. The parietal pelvic peritoneum is transected from the muscles and fascia down towards the intraperitoneal space. The inferior limits of the dissection are marked by the boundaries formed by the broad ligament (the mesoovarium, the mesosalpinx, and the mesometrum), the mesorectum, and the mesocolon.
9.  Extraperitoneal cutting of the broad ligament
  The round ligaments are divided as they enter the internal inguinal ring.
10.  Central ligation of the uterine arteries
  Uterine arteries are ligated laterally to the ureter at their origin, the anterior division of the internal iliac artery (marginal ligation). This ligation enables the excision of the entire parametrium in one block with the main specimen.
11.  Dissection of the uterus at the level of the fornices of the vagina
  The lower aspect of the uterus is dissected from the vagina, and the vaginal cuff is closed.
12.  Dissection of the rectum
  The rectovaginal septum is exposed. The perirectal fat is divided 2–3 cm beneath the peritoneal reflection so that the portion of the tumor occupying the cul-de-sac is removed intact with the specimen. The rectal musculature is skeletonized using electrosurgery so that a stapler/purse-string can be used to close the rectal stump. Finally, the rectum is divided, and the cancer specimen is removed.
13.  Creation of a colorectal anastomosis
  The proximal colon is anastomosed to the rectal remnant with a stapling device.
  *End-to-end*: A circular stapling device is passed into the rectum, and the trochar penetrates the staple line. A purse-string applier is used to secure the stapler anvil in the distal descending colon. The body of the circular stapler and anvil are mated, and the stapler is activated to complete the low colorectal anastomosis.
  *Side-to-end*: The purse-string clamp is applied at the inferior level of the resection. A straight needle is passed through the clamp to form purse-string sutures, which are used to secure the staple anvil in the rectal stump.
  The lumen of the colon is visualized and progressively widened using three dilators of increasing diameters (25, 28, and 31 mm). The EEA instrument is inserted through the end of the colon, and the trochar penetrates the sidewall of the bowel. The body of the circular stapler and anvil are mated, and the stapler is activated. The circular stapler is then removed, and the distal end of the colon is closed below the colorectal anastomosis with a linear stapler TA 55.
14. Leak testing

### Of colorectal anastomosis

To evaluate the stapled colorectal anastomosis, the proximal and distal tissue rings are examined for completeness.

Air leak testing is conducted, and air is insufflated into the rectum with a water-filled pelvis to check for an airtight circle of staples. Two hands should easily pass beneath the sigmoid colon to ensure no tension at the stapled anastomosis. A rectal examination is conducted to check for staple-line bleeding at the anastomosis.

### Of the bladder

The integrity of the bladder is analyzed with an intraoperative methylene-blue test (IMBT).

*The division of the colon is delayed, while a greater omentectomy is conducted first. This order of operations enables an assessment of the “oncological cleanliness” of the entire colon to select the optimal place for cutting. The splenic flexure and the transverse colon are frequently involved, which involvement is hidden by the metastatic omentum.

## Results

The median age of patients was 58 years (range: 36–80 years). The median body mass index (BMI) was 25.7 (range: 21–30). High-grade serous ovarian cancer (HGSOC) was the predominating histological type and was detected in 15 of 20 (75 %) cases. The most common FIGO stage was IIIC confirmed in 50 % of patients. Most cases presented with ascites exceeding 1000 ml (80 %). Detailed data of the pre-treatment and disease-related characteristics are shown in Table 2.

**Table 2:** Patients characteristics

Feature	Value/no. of patients
Median age years	58 years (range 36–80)
Median BMI	25.7 (range 21–30)
ASA score^a^	
1	6
2	11
3	3
Tumor histology	
HGSOC	15
LGSOC	1
Endometroid	2
Clear cell	1
Musinous	1
FIGO stage^b^	
II B	2
III B	3
III C	10
IV A	5
Ascites	
<1000 cm^3^	4
>1000 cm^3^	16

^a^American Society of Anesthesiologists Classification of Physical Status

^b^International Federation of Gynecology and Obstetrics

**Fig. 2:**
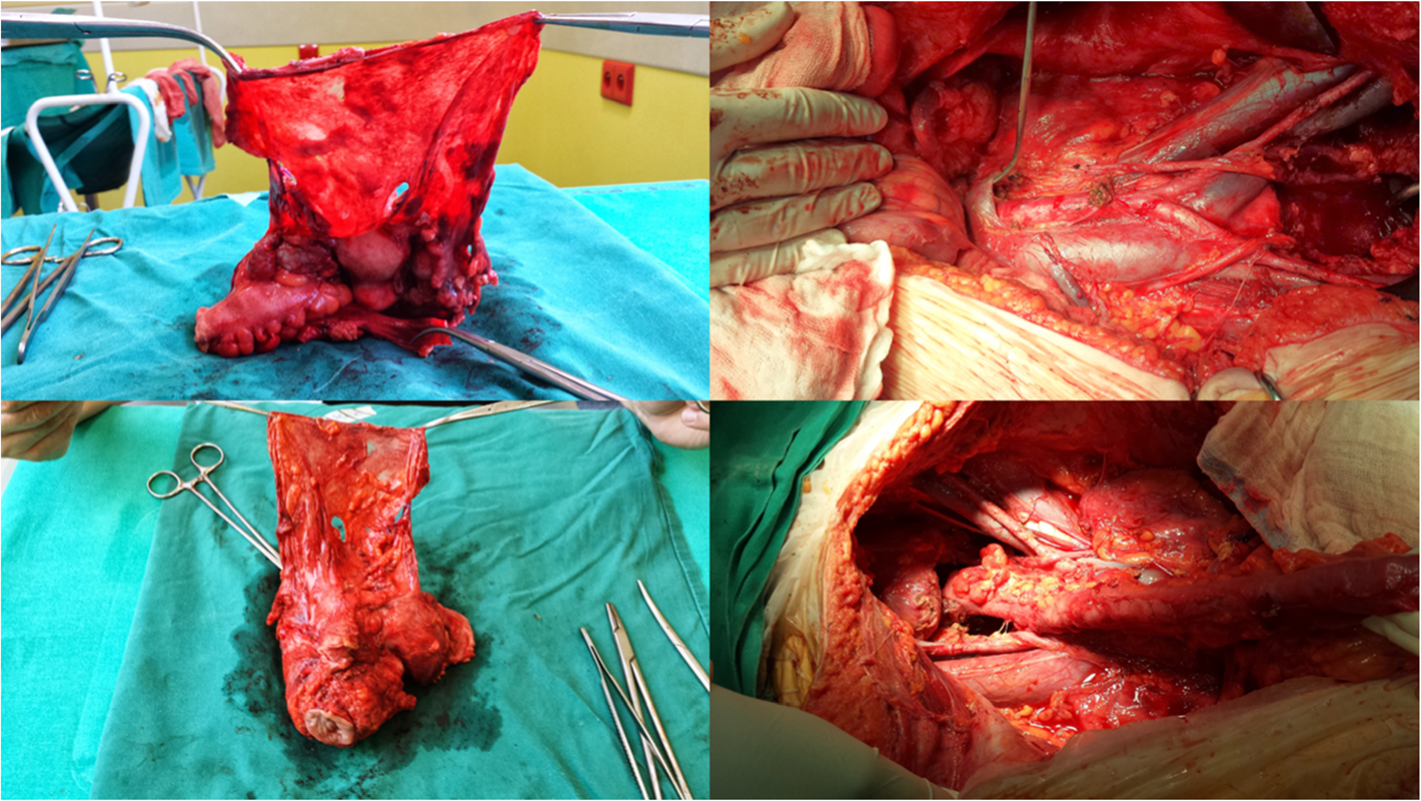
Intraperitoneal cancer compartment (including the infiltrated part of the bowel) and oncological clearness following the modified en block pelvic resection

In all cases, no gross residual disease was achieved. In all cases, histopathology results also confirmed bowel infiltration of the cancer cells with the involvement of mesenteric lymph nodes in 11 cases (55 %). Operative details are shown in Table 3. Samples of the removed tumor bed and the oncological clearness of the pelvis after surgery are shown in Fig. 2. The median duration of the procedure and hospital stay was 320 min (range: 205–430 min) and 12 days (range: 7–44 days), respectively. The median estimated blood loss (EBL) was 600 cm^3^ (range: 400–1000 cm^3^). Nineteen patients received blood transfusions (99 %). The median number of red blood cell (RBC) transfusion units was 4 (range: 0 to 6 units). Six of 20 patients (30 %) required postoperative intensive care. The median number of days spent by these patients in the intensive care unit (ICU) was 4 (range: 1–6 days). Four patients received total parenteral nutrition (20 %).

**Table 3:** The visceral resections and parietal peritonectomy procedures conducted during primary debulking surgery (PDS)

Type of peritonectomy	Provided resections	No. of patients
Pelvic peritonectomy	Uterus, ovaries, and rectosigmoid colon	20
Left upper quadrant peritonectomy	Greater omentectomy	20
	Splenectomy	4
	Diaphragm: stripping/resection	1
Right upper quadrant peritonectomy	Tumor on Glisson’s capsule of the liver	1
	Diaphragm: stripping/resection	4
Anterior parietal peritonectomy	Old abdominal incisions, umbilicus	0
	epigastric fat pad	0
Omental bursectomy	Gallbladder and lesser omentum	0
Type of lymhadenectomy	Selective PALND/PLND	10
	Systematic PALND/PLND	2

One patient experienced a complication associated with en bloc resection, namely an anastomosis site leak. This patient accordingly underwent further surgery during the same admission. The anastomosis site leak was managed with a diversion colostomy and pelvic cavity drainage. The colostomy was reversed after chemotherapy was complete. There were no surgery-related deaths; however, one woman died 12 days post-surgery. Her death was due to pulmonary embolismus, though all 20 patients received combined pharmacologic and mechanical methods of prophylaxis of venous thromboembolism (VTE) during hospital stay.

All surviving patients (*n* = 19) received platinum-based chemotherapy. The median number of days between the primary surgery and the first cycle of chemotherapy was 48 days (range: 20–147 days). The median follow-up time period was 19 months (range: 8–31 months). No patient experienced a recurrence of disease during the time of observation. Detailed data on the course of treatment for particular cases are shown in Table 4.

**Table 4:** Modified en block pelvic resection—course of treatment

Age years	Surgery duration minutes	EBL cc	ICU days	RBC units	PN	Surgical complication	Hospital stay days	Time between PDS and Cht days
62	205	400	0	2	0	0	7	32
58	310	600	0	4	0	0	13	31
38	350	900	3	5	0	0	9	44
62	350	400	0	4		Anastomosis leak	25	48
36	280	700	0	4	0	0	7	51
74	210	400	0	0	0	0	12	62
80	270	1000	5	6	1	0	12	x death
65	380	650	5	4	1	Wound infection	44	147
64	345	400	5	1	1	0	12	52
64	310	600	0	4	1	0	18	48
52	305	450	0	1	0	0	13	48
55	410	400	0	2	0	0	9	35
66	320	550	0	3	0	0	15	61
54	300	700	0	5	0	0	11	53
55	430	950	1	5	0	0	12	26
72	320	400	0	2	0	0	12	31
48	345	600	0	4	0	0	9	56
59	300	800	0	6	0	0	12	60
46	355	500	0	3	0	0	8	20
54	345	700	1	4	0	0	20	25

*EBL* estimated blood loss, *ICU* intensive care unit, *RBC* red blood cell, *PN* parenteral nutrition, *PDS* primary debulking surgery, *Cht* chemiotherapy

## Discussion

The conventional technique procedure prioritizes the mobilization of the tumor-bearing segment followed by central vascular ligation and the ligation of other portions of the vasculature. Conversely, the no-touch isolation technique prioritizes central vascular ligation, followed by the mobilization of the tumor-bearing segment [9].

In advanced EOC, multi-visceral and peritoneal metastases create a tumor-bearing segment supplied by the extraperitoneal vascular system.

It is well-known that a complete peritonectomy and visceral resections are required to achieve a complete clearance of pan pelvic disease in these patients. The preponderance of data suggests that colon resection to achieve optimal cytoreduction positively impacts survival rates [15].

To achieve cytoreduction of microscopic residual disease in disseminated EOC cases consistent with the requirements of cancer no-touch isolation, a modified en bloc pelvic resection was proposed. This modified resection aims to initially reach the retroperitoneal space where the central vascular ligation is performed.

In the first step of the operation, the peritoneum is incised along the lateral peritoneal reflections immediately medial to the white line of Toldt from the left and right paracolic sulci cephalad to the splenic and hepatic flexures of the colon. The mesentery of the left and right colons is divided from all its retroperitoneal attachments. This dividing not only enables the identification of the ovarian and mesentery vessels but also elevates the entire colon with metastatic omentum, thus allowing the affliction of the upper abdomen with cancer to be properly assessed.

It has been suggested that the inefficiency of upper abdomen surgery limits optimal cytoreduction [16]. Hence, this surgical step could reveal all potentially unresectable lesions and is therefore proposed to initiate all aggressive surgical plans to eradicate EOC.

The left and right ovarian vessels are dissected and ligated at the level of the left renal vein and the vena cava, respectively.

Central vascular ligation of the tumor-bearing segment of the colon is performed. From a practical perspective, this ligation should be proceeded by the opening of the omental bursa to assess the “oncological cleanliness” of the colon.

The surgical plan includes central lymphovascular ligation and retroperitoneal lymph node dissection (LND). LND is a questionable procedure to use in cases of advanced ovarian cancer. We aimed to excise enlarged, suspicious nodes during the cytoreduction or to perform a systematic lymphadenectomy in cases with cancer spread macroscopically limited only to the pelvis. Current prospective studies such as the LION study (AGO-Ovar) will confirm the appropriateness of using LND in EOC. Notably, none of the 12 cases showed postoperative lymphocele with the lymph node dissection, which result could indicate another positive impact of performing the central lymphovascular ligation prior to excising enlarged, suspicious nodes.

The next proposed modification entails an excision of the entire segment of affected peritoneum, including areas without visible lesions. For example, if the peritoneal metastases are present in the pelvis, a complete pelvic peritonectomy is conducted. Recently, radical surgery together with hyperthermic intraperitoneal chemotherapy (HIPEC) as described by Sugarbaker [14] has been widely promoted for use in EOC cases.

Sugarbaker did not excise uninvolved surfaces of the peritoneum because he believed that microscopic disease, if unaddressed, will be eradicated by HIPEC performed immediately after surgery. Existing evidence suggests that primary debulking surgery followed by HIPEC has an unexpectedly negative impact on prognosis in EOC [17]. The results of ongoing randomized control trials will be published not sooner than in the next 3 years. Therefore, the approach of excising the entire segment of involved peritoneum, rather than performing experimental perioperative intraperitoneal chemotherapy, seems reasonable, particularly given the lack of observed complications related to the excision in our cohort.

### Rationale for modification

The impact of no tough isolation technique on intraoperative cancer dissemination will be difficult to prove. However, the proposed central vascular ligation should, if not decrease intraoperative cancer dissemination, at least decrease complications such as blood loss and lymphocoele formation. Elevating the entire colon while opening the retroperitoneal space seems to be highly reliable for predicting optimal cytoreduction. Excising the entire segment of involved peritoneum, including areas without visible lesions, is safe and could be a cost-effective alternative for HIPEC.

### Course of treatment

The duration of surgery and hospitalization was comparable to the respective durations reported in other studies on en block pelvic resection published after 2000 [18–24]. Patients in our cohort had a lower EBL and the highest rate of complete cytoreduction compared with other reports [18–24]. We believe that the decrease in blood loss is caused by the introduction of central vascular ligation and by the fact that surgery is starting from the healthy tissues surrounding the tumor compartment. It was proven that the tumor-bearing segment has increased vascularization caused by cancer neoangiogenesis [25]. A surgical operation conducted through an anatomically unchanged (i.e., healthy) compartment could also be responsible for the increased resectability of ovarian cancer tissue.

We observed anastomosis leakage in 1 of 20 cases (5 %). Only in one recent study was the percentage of this complication is higher (8.7 %) [23]. Remaining studies published after 2000 show lower rates of anastomosis leakage, namely 0.8 %, [19] 1.7 % [21], 2.2 % [22], 3 % [24], 3.2 % [20], and 4.5 % [18]. All studies on en block pelvic resection were conducted with a limited number of cases, and the subsequent results of the studies do not reflect the real (average) rate of the anastomosis leakage of colorectal anastomosis. A meta-analysis of randomized control trials (RCTs) of colorectal anastomosis surgery published in 2001 revealed that the overall stapled clinical leak rate is 6.3 % [26]. Given these data, our results (5 %) seem fully acceptable.

The single complication observed in our study could be explained by the poor nutritional status of the patient. We routinely analyzed serum albumin levels prior to surgery, yet this method appeared to be ineffective in identifying patients with severe nutritional risk. Since our study, we have incorporated an assessment of nutritional status based on the European Society of Parenteral Nutrition (ESPEN) Guidelines on Enteral Nutrition [27] into our qualification process. Currently, all patients with severe nutritional risk receive parenteral nutrition for 10–14 days prior to surgery even if surgery must be consequently delayed.

Recent studies on stapled colorectal anastomosis have indicated *side-to-end* anastomosis as safer than *end-to-end* anastomosis [28]. *Side-to-end* anastomosis tends to fill the pelvis, reducing the area of dead space in which a hematoma or collection of hematomas could develop [29].

Indeed, our observed case of leakage was one of two cases in which *end-to-end* colorectal anastomosis was created, which could thus be additional reason for the complication.

There were no surgery-related deaths; however, one woman died due to pulmonary embolismus 12 days post-surgery. She received combined pharmacologic and mechanical methods of prophylaxis of VTE during her hospital stay. Post mortem, it was found that she had been a mutation carrier of factor V Leiden. This fact was unpredictable; thus, we believe that her death should not affect the appraisal of the described surgical procedure.

All surviving patients received six courses of platinum-based chemotherapy.

The time interval from surgery to the start of chemotherapy was 48 days (range: 20–147 days). This interval is longer than the average period of 4–5 weeks reported in the literature [30]. This delay has been suggested as impairing a patient’s prognosis [31]. Recently, however, it was demonstrated that the timing of postoperative chemotherapy did not influence the overall survival rate in women without postoperative residual disease [30]. Indeed, none of our optimally debulked patients experienced a recurrence of the disease during the time of our observation. The median follow-up occurred only 19 months following surgery (range: 8–31 months). PFS periods reported in the literature range from 14 to 18 months [32]; hence, we believe that an en bloc pelvic resection preceded by a central ligation of the vessels supplying the tumor bed could potentially improve the outcome of ovarian cancer patients.

Prospective clinical trials with a control group are needed to assess the exact oncological benefit of the presented approach.

### Summary of proposed modification

The tumor-bearing segment (pelvis) is not touched. In the first step, a retroperitoneal space is opened by incising along the lateral peritoneal reflections immediately medial to the white line of Toldt from the left and right paracolic sulci cephalad to the splenic and hepatic flexures of the colon. This method enables the vessels supplying tumor bed to be reached and ligated first (ovarian and mesenteric anterior vessels) as well as the lymph nodes involvement to be assessed. In advanced cases (including omental cake), this step should be proceeded by opening the omental bursa to assess the “oncological cleanliness” of the colon. Mobilization of the entire colon allows a proper determination of the affliction of the upper abdomen by cancer. Finally, intraperitoneal cancer compartment (including the infiltrated portion of the bowel) is surrounded from the outside (healthy tissues) by separating the entire peritoneum (not merely the involved portion) from the bladder and the pelvic wall and then resecting in one block. A colorectal anastomosis is created. In our cohort of 20 ovarian cancer patients, this new approach resulted in low blood loss and a high rate of optimal cytoreduction.

## Conclusions

Performing a central ligation of vessels supplying the tumor bed prior to an en bloc pelvic resection is feasible with acceptable morbidity and mortality rates. The impact of this modification on intraoperative cancer spread and the decrease in blood loss as well as oncological outcome should be confirmed in future studies.
